# Detection of Mercury in Cases of Poisoning

**Published:** 1869-04

**Authors:** 


DETECTION OF MERCURY IN CASES OF POISONING.—This is a point of much importance to medical men and to professional toxicologists. The following method was recently employed by M. Buchner in a case of poisoning with corrosive sublimate. The organic remains having been disintegrated by a hot mixture of chlorate of potash and hydrochloric acid, the solution was diluted and saturated with sulphuretted hydrogen. After the lapse of some hours, the sulphide formed was collected, dissolved in aqua regia, and reduced by evaporation to a small volume. A little water being added, a bright piece of copper wire is placed in the liquid; and when mercury is present the wire becomes grey, at the latest, in two days. The copper is withdrawn, dried between folds of blotting-paper, and heated in a wide test tube. The mercury is more easily distinguished by removing the wire, and placing in the tube a drop of tincture of iodine. M. Riederer, having remarked that the sulphide of mercury which is formed by this process always contains organic matter, has recourse to dialysis. He operates in the following manner. After disorganization by chlorate of potash and hydrochloric acid, the mercury in solution is precipitated by sulphuretted hydrogen, the sulphide collected dissolved in a mixture of chlorate of potash and hydrochloric acid, and dialysed with 500 c.c. of water. At the end of five days, the water is evaporated, and the dialysis repeated. After this treatment, the solution is again saturated with sulphuretted hydrogen; the precipitate is washed with ammonia and sulphide of ammonium, then with weak nitric acid, and finally treated afresh with hydrochloric acid and chlorate of potash. Operating upon dogs with calomel, M. Riederer has recognised that the greater part of the mercurial compound is eliminated by the excrements, and that, for the rest, more collects in the liver than in the muscles.—Paris correspondent of *Chemical News*, January 15th.

